# Difluorovinyl Liquid Crystal Diluters Improve the Electro-Optical Properties of High-∆*n* Liquid Crystal Mixture for AR Displays

**DOI:** 10.3390/molecules28062458

**Published:** 2023-03-08

**Authors:** Jiaxing Tang, Zihao Mao, Zhongwei An, Ran Chen, Xinbing Chen, Pei Chen

**Affiliations:** 1 International Joint Research Center of Shaanxi Province for Photoelectric Materials Science, Key Laboratory of Applied Surface and Colloid Chemistry (MOE), Shaanxi Key Laboratory for Advanced Energy Devices, Shaanxi Engineering Laboratory for Advanced Energy Technology, School of Materials Science and Engineering, Shaanxi Normal University, Xi’an 710119, China; 2 Xi’an Modern Chemistry Research Institute, Xi’an 710119, China

**Keywords:** high birefringence liquid crystal, diluter molecule, AR displays, large dielectric anisotropy, low viscosity

## Abstract

A liquid crystal (LC) mixture in liquid crystal on silicon (LCoS) is the core material for augmented reality (AR) displays. However, a LC mixture with high birefringence (Δ*n*) and large dielectric anisotropy (Δ*ε*) possesses high viscosity (*γ*_1_), which results in a slow response time of LCoS devices for AR displays. This work proposes to apply difluorovinyl-based LC diluters to fine balance the low viscosity, high ∆*n*, and large ∆*ε* of the LC mixture for a fast response time. Through studying their effects on the key electro-optical properties of a high-∆*n* LC mixture, it is found that doping these diluter molecules to a high-∆*n* LC mixture can decrease the viscoelastic coefficient (*γ*_1_/*K*_11_), increase ∆*ε* and the figure of merit, maintain a wide nematic phase temperature range, a high clearing point, and ∆*n*. It also means that these diluters could effectively regulate the relationship between ∆*n*, ∆*ε*, and *γ*_1_ in the LC mixtures to achieve a fine balance of various excellent properties and further improve the LC device’s response time. The widespread applications of these liquid crystal diluters in emerging liquid crystal optical devices are foreseeable.

## 1. Introduction

Liquid crystal on silicon (LCoS) devices not only have performance advantages of excellent optical modulation ability [1,2], high resolution [3], high brightness [4], and low driving voltage, but can also expand the field of view, suppress chromatic aberration, and improve the frame rate. Therefore, they have widespread applications in augmented reality (AR) displays [5,6,7]. The electro-optical properties of fast-response liquid crystal (LC) materials used in LCoS devices depends on many factors, such as birefringence (∆*n*) [8,9], dielectric anisotropy (∆*ε*) [10,11,12], elastic constants, and rotational viscosity (*γ*_1_) [13,14,15]. To achieve an ultra-high resolution LCoS device, one of the most effective methods is to employ high-∆*ε* LC materials [16] to reduce the required driving voltage. The high-∆*n* LC materials can reduce the thickness of LC cells and inhibit the fringing field effect [17] in LCoS devices. Low viscosity LC materials can effectively improve the response time to overcome the discomfort caused by the vergence-accommodation conflict in AR displays. Consequently, it is particularly important to develop LC materials with large ∆*ε*, high ∆*n*, and low viscosity for AR displays.

Generally, various LC compounds with excellent comprehensive properties need to be mixed into new LC mixtures, which mainly include a high-∆*n* component, a large ∆*ε* component, a low-viscosity component, etc. [18]. Tolane liquid crystals as the representative of large π-conjugated structures [19,20] show the performance advantages of high ∆*n* and low viscosity, which are suitable as high-∆*n* components. The lateral fluorine substituent not only decreases the melting point and increases the ∆*ε* [21], but is also utilized to widen the nematic phase temperature range of LCs [22]. Accordingly, fluorinated liquid crystals are usually optimized as large ∆*ε* components. In the representative structures with low viscosity, the two-ring structures based on the cyclohexyl skeleton show low viscosity and low melting point, thereby they are preferred as low-viscosity components. Unfortunately, LC mixtures with high ∆*n* and large ∆*ε* possess strong intermolecular interactions, which inevitably lead to an increase in rotational viscosity. LC diluters [23,24,25] have been reported to effectively reduce the viscosity of LC mixtures used in TFT displays. For example, LC diluter molecules **3HHV** (Δ*n* = 0.029), **CP53** (Δ*n* = 0.033), and **CC53** (Δ*n* = 0.040) have been reported in previous references [26,27]. However, most reported LC diluters are based on the cyclohexyl skeleton and display low Δ*n* and poor compatibility, which are not suitable for high-Δ*n* LC mixtures. As a result, it is important to develop new high-Δ*n* LC diluters to improve the performance of LC optical devices. Furthermore, we expect to employ these diluters to effectively regulate the relationship between ∆*n*, ∆*ε*, and *γ*_1_ in the LC mixtures to achieve a fine balance of various excellent properties and further improve the LC device’s response time.

**Figure 1:**
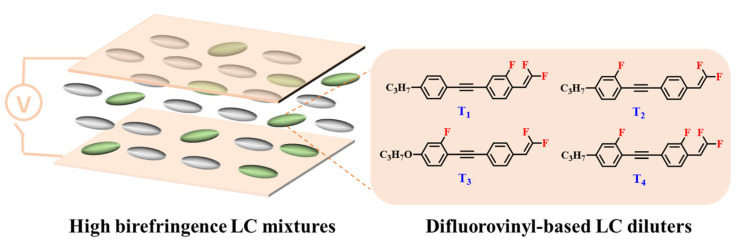
Chemical structures of difluorovinyl-based LC diluters and schematic diagram of high birefringence LC mixtures.

Against the above background, this work pays attention to the research on a series of difluorovinyl LC diluters as LC dopants to improve the electro-optical properties of high-∆*n* LC mixtures. Their molecular structures and corresponding schematic drawings in LC mixtures are shown in Figure 1. Firstly, we selected one commercial high-∆*n* LC mixture (HTD028200-100) as the parent formulation and studied their solubility in this parent mixture to further reveal their effects on the low-temperature storage performance and clearing point of the parent LC mixture. Secondly, we focused on the influence of four new LC diluters on the key electro-optical properties of the high-∆*n* LC mixture, such as ∆*n*, ∆*ε*, and viscoelastic coefficient (*γ*_1_/*K*_11_). Finally, the comprehensive properties of these LC diluters in the high-∆*n* LC mixture were further evaluated by using a figure of merit (FoM) parameter. Research results show that adding each of such diluters to one high-∆*n* LC mixture can effectively reduce the viscosity, increase the dielectric anisotropy, and maintain high birefringence, which is conducive to fast-response LC optical devices.

## 2. Results and Discussion

### 2.1. Phase Transition Temperature

The operating temperature of the LCoS devices depends on the LC phase temperature range of the LC materials. Previous studies [27,28] have found that the addition of the LC diluter can effectively reduce the melting point of LC mixtures, but often can also significantly reduce its clearing point, which is unfavorable to the wide temperature range of LC mixtures. The DSC instrument was employed to measure the phase transition temperatures of four LC diluters **T_1_**–**T_4_** and their corresponding LC mixtures **H_1_**–**H_4_**. The test results are summarized in Table 1.

Firstly, we stored the mixtures **H_1_**–**H_4_** and the parent mixture **HTD** in an ultra-low temperature refrigerator and observed them to find that their low-temperature storage temperatures were all lower than −15 °C. As shown in Table 1, the new LC mixtures **H_1_**–**H_4_** formed by the parent mixture with each of four diluters can maintain wide nematic phase temperature intervals of over 100 °C. As the number of fluorine atoms on the LC diluter molecules increases [29], the clearing points of the corresponding LC mixtures decrease. By comparing the clearing points of mixtures **H_1_**–**H_4_**, it is found that the lateral fluorine substituent on the first benzene ring of the diluter is more conducive to improving the clearing point of the parent mixture **HTD** than that with the lateral fluorine substituent on the second benzene ring. Among all LC mixtures, the LC mixture **H_3_** has a higher clearing point, which increases by 0.1 °C relative to that of **HTD**, which is attituded to the high clearing point of the diluter molecule **T_3_**. This high clearing point means that the corresponding LC mixture would occur with a small fluctuation of temperature-dependent performances. According to the Schroder-Van Laar equation, the clearing point of nematic LC satisfies a linear add-up relationship [27]. The extrapolated clearing points of these four LC diluters are 50.8 °C, 66.8 °C, 94.8 °C, and 21.8 °C, respectively, which are 4.96~8.71 °C different from the directly measured *T*_C_^1^ results (DSC data). Among these four mixtures, the clearing point of mixture **H_2_** is closer to the extrapolated value, which is more conducive to the nematic potential of the parent LC mixture [27].

### 2.2. Birefringence

**Figure 2:**
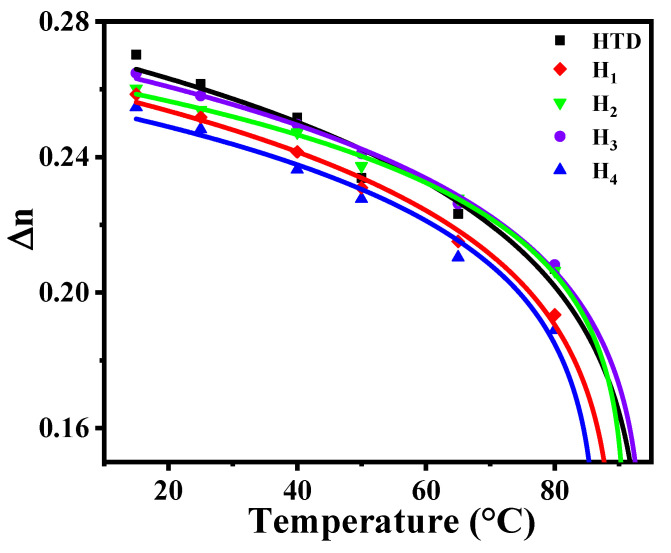
Temperature-dependent birefringence at *λ* = 633 nm.

LCoS devices [2,5,16] can easily realize both intensity modulation and phase modulation, and they are widely used in AR displays, phased array devices, laser beam modulation, terahertz modulation, and other LC optical devices due to their excellent phase modulation characteristics. The phase modulation characteristics of LCoS devices mainly depend on the birefringence performance of LC materials, and the required phase change *δ* (*δ* = *2πd*∆*n*/*λ*) is 2π. Herein, we studied the effects of four LC diluters on the ∆*n* of the parent LC mixture in response to temperature and wavelength. We filled these LC mixtures into the corresponding LC cells with the same thickness of 5.16 μm and then placed them on one Linkam THMS600 hot-stage. Using a He-Ne laser (*λ* = 633 nm) as the light source, we measured the ∆*n* of these LC mixtures at temperatures of 10~80 °C. Figure 2 displays the temperature-dependent birefringence curves of these LC mixtures, where points represent the test data and solid lines represent the curves fitted by Equation (2).

After doping diluters, the new LC mixtures can still maintain high birefringence (∆*n* ≥ 0.248 at 25 °C @ 633 nm), and their ∆*n* values decrease sequentially: **H_3_** > **H_2_** > **H_1_** > **H_4_**. Obviously, the largest birefringence value (0.258) of **H_3_** is equivalent to the ∆*n* value of the parent LC mixture, meaning that the diluter structure **T_3_** has the least influence on the birefringence of the system. This is mainly because the *n*-propoxy terminal group in the LC diluter **T_3_** has a greater molecular polarizability than the corresponding *n*-propyl terminal, thus increasing the molecular polarizability of the LC diluters, resulting in a larger ∆*n*. As the temperature increases, the ∆*n* values of these four LC mixtures decrease at roughly the same rate, and then the ∆*n_0_* and *β* parameters were extrapolated by Equation (2); the corresponding results are summarized in Table 2.

**Figure 3:**
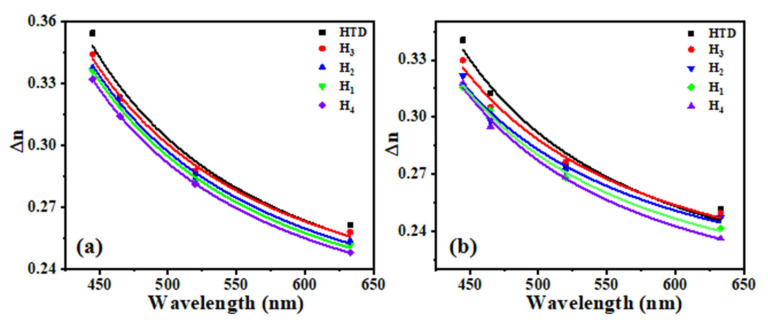
Wavelength-dependent birefringence at T = 25 °C (**a**) and T = 40 °C (**b**).

In addition, to further study the electro-optical performance at different wavelengths, we used tunable argon ion lasers (*λ* = 445 nm, 465 nm and 520 nm) and a He-Ne laser (*λ* = 633 nm) as light sources to test their ∆*n* values under different conditions. Figure 3a,b show the wavelength-dependent birefringence curves at 25 °C and 40 °C, respectively, where points represent test data and solid lines represent curves fitted through Equation (3). The results are summarized in Table 2. With the increase in wavelength, the ∆*n* value of each LC mixture decreases. At the same wavelength, their ∆*n* values decrease sequentially: **H_3_** > **H_2_** > **H_1_** > **H_4_**. Among diluters **T_2_**–**T_4_**, diluter molecule **T_4_** shows a greater influence on the ∆*n* value of the LC mixtures due to the significant effects by the lateral fluorination.

### 2.3. Viscoelastic Coefficient

**Figure 4:**
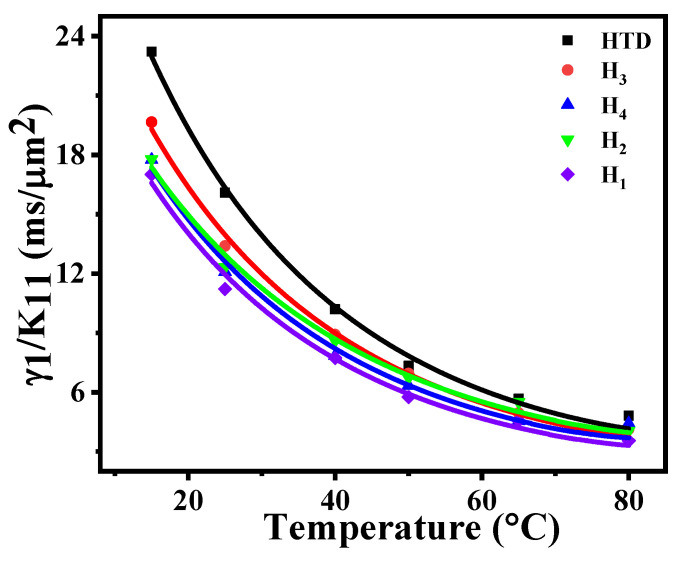
Temperature-dependent visco-elastic coefficient at *λ* = 633 nm.

The fast-response speed of liquid crystals is mainly associated with their properties of low viscoelastic coefficient and high Δ*n*. Therefore, we investigated the effects of these LC diluters on the viscoelastic coefficient of the parent LC mixture, which we expected to lay the theoretical foundation for designing low-viscosity LC mixtures. Using a He-Ne laser (*λ* = 633 nm) as a light source and LC cells with the same thickness of 5.16 μm, the temperature-dependent viscoelastic coefficients (*γ*_1_/*K*_11_) of these LC mixtures were measured and obtained. As shown in Figure 4, the points represent the measured data, and solid lines represent curves fitted through Equation (4). Then the corresponding *E_a_* and *A* parameters were extrapolated, and their results are summarized in Table 3. In comparison with these four diluters, diluter **T_2_** has the greatest influence on the viscoelastic coefficient value of the LC mixture. At 25 °C, the viscoelastic coefficients of the LC mixtures **H_1_**–**H_4_** decrease by 16.8%, 28.8%, 23.4%, and 24.9%, respectively. At 40 °C, the viscoelastic coefficients of **H_1_**–**H_4_** decrease by 12.6%, 24.2%, 15.8%, and 23.1%, respectively. Initially, the viscoelastic coefficients of these four LC mixtures decrease rapidly with the increase in temperature, and then tend to be flat when approaching their clearing points. Compared with **HTD**, the activation energy *E_a_* values of **H_1_**–**H_4_** were all reduced (*E_a_* ≤ 257.7). The smaller the activation energy value is [30], the faster the low-temperature response speed is. This means that the LC diluters can effectively reduce the viscoelastic coefficient [31] of the parent LC mixture at low temperature, which may be beneficial to improve the low-temperature response time of LC mixtures.

### 2.4. Dielectric Anisotropy

**Figure 5:**
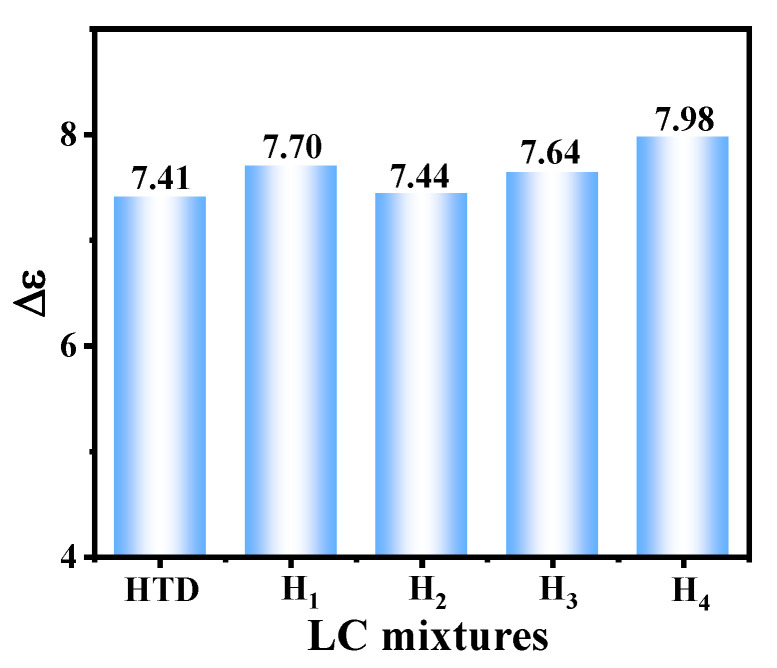
Comparison for dielectric anisotropy of LC mixtures.

The electric field response performance of LC mixtures mainly depends on the dielectric anisotropy and elastic constant of LC materials. As we know, there is little research on the regulation of elastic constants. Liquid crystal chemists [21,32,33] often employ various molecular engineering strategies to synthesize many LC compounds with large ∆*ε*, then LC physicists utilize these LC compounds to prepare LC mixtures with large ∆*ε* [34]. These large-∆*ε* LC mixtures can be used to reduce the working voltage of LC devices, meanwhile realizing the energy savings and consumption reduction in all kinds of LC devices. Herein, we studied the effects of these new LC diluters on the dielectric anisotropy of the parent LC mixture. The ∆*ε* test results of all LC mixtures at 25 °C are listed in Figure 5. Compared with **HTD**, the ∆*ε* values of the new LC mixtures doping with diluters are all enhanced, and the corresponding ∆*ε* values of mixtures **H_1_**–**H_4_** are 7.70, 7.44, 7.64, and 7.98, respectively. Compared with **H_1_** and **H_2_**, when the lateral fluorine atom is located on the second benzene ring of the diluter molecule, the larger dipole moment of **T_1_** promotes the ∆*ε* value of **H_1_** to increase. Among all LC mixtures, mixture **H_4_** has the highest ∆*ε* value (7.98), which increases by 7.7% compared with that of **HTD**, which is due to the fact that diluter **T_4_** contains four fluorine atoms on the molecule. Our experimental results are completely different to previous results [27,28] about the effect of reported LC diluters on the ∆*ε* values of LC mixtures. The above results show that these difluorovinyl-based LC diluters can increase the ∆*ε* value of the parent LC mixture, which will help reduce the operating voltage of LC devices, thus improving the response time.

### 2.5. Voltage-Dependent Phase Change (V-Φ) Curves

**Figure 6:**
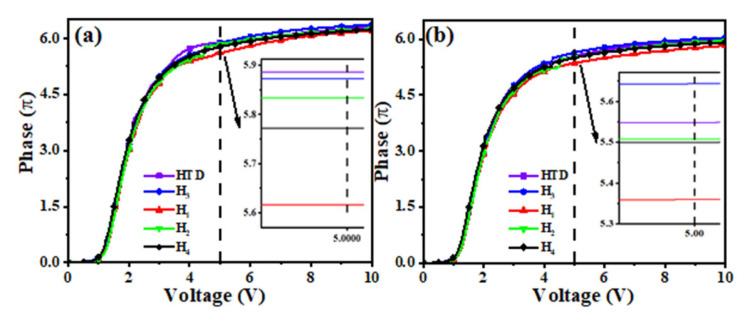
Voltage-dependent phase (*V-Φ*) curve at 25 °C (**a**) and 40 °C (**b**).

LCoS devices show advantages of 2π phase modulation in AR displays. With the limitation of the maximum driving voltage for the LCoS backplane, we selected 5 V as the maximum operating voltage to achieve 2π phase modulation. To study the voltage-dependent phase change in these LC mixtures in real LCoS devices, four new LC mixtures and a parent LC mixture were injected into LC cells with the same thickness of 7.86 µm. Due to the influence of backplane driving circuits and the employed light source, the operating temperature of the LCoS devices was 40~60 °C [17]. Therefore, we tested the voltage-transmittance (V-T) curves of all LC mixtures at the temperature of 40 °C and the operating wavelength of 633 nm. As the reference temperature, the V-T curves at 25 °C were also tested. The measured V-T curves were transformed into voltage-dependent phase (V-Φ) curves, and the results are shown in Figure 6. With the increase in temperature, the phase change gradually decreases, which is mainly due to the decrease in ∆*n*. At 40 °C, all LC mixtures can maintain a larger phase change (5.36~5.64π) at the voltage of 5 V, and the phase modulation change in **H_3_** is the largest one. According to the formula *δ* = *2πd*∆*n*/*λ*, we can realize the required 2π phase modulation by reducing the thickness of the LC cell when using the LC mixtures mentioned above, while increasing the response time of LC devices.

### 2.6. Figure of Merit

**Figure 7:**
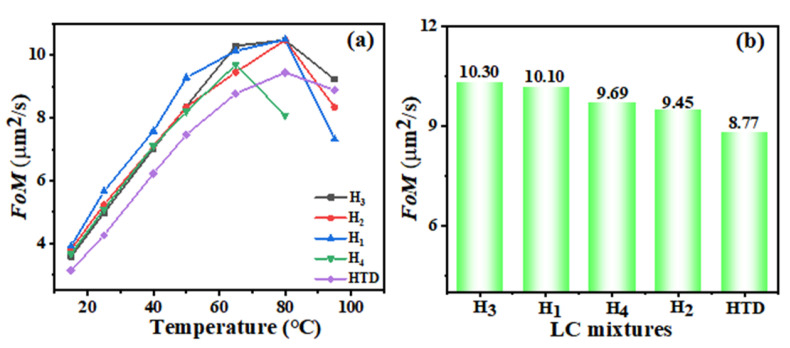
Temperature-dependent FoM of LC mixtures (**a**), and comparison for the FoM of LC mixtures at 65 °C (**b**).

The FoM parameter is a representation of the response time due to the fact that it takes into account both the birefringence and viscoelastic coefficient [31]. The larger the FoM value is, the better the response performance of the LC materials is. To better study the response performance of LC materials, Figure 7a describes the temperature-dependent FoM of various LC mixtures. With the increase in temperature, the *FoM* values of the LC mixtures increase. As the temperature approaches the clearing point, the *FoM* dropped sharply, which is mainly due to the sharp decrease in both Δ*n* and *γ*_1_/*K*_11_. Except for mixture **H_4_**, the best temperature of the other four LC mixtures to obtain the largest FoM value is about 80 °C. Below the temperature to 80 °C, the FoM values of the new LC mixtures **H_1_**–**H_4_** are higher than that of **HTD**, meaning that the introduction of such LC diluters is conducive to improving the response time of LC mixtures. The temperature at the largest FoM value of **H_4_** is 65 °C, therefore we further compared the FoM values of five LC mixtures at 65 °C (Figure 7b). The results show that the FoM values corresponding to **HTD**, **H_1_**–**H_4_** are 8.8, 10.1, 9.5, 10.3, and 9.7, respectively. Among them, the mixture **H_3_** possesses the largest one, and the value is 17% larger than that of **HTD**, which is mainly due to its larger Δ*n*.

### 2.7. Discussions

Table 4 summarizes the physical properties of four LC mixtures doping with our difluorovinyl LC diluters at 25 °C. It can be seen from Table 4 that these new LC mixtures all show wide LC phase temperature ranges, and the LC diluters have little influence on the clearing point of **HTD**. Among them, mixture **H_4_** has the lowest clearing point due to the addition of diluter **T_4_** containing more lateral fluorine atoms. LC mixture **H_3_** doping with diluter **T_3_** terminated with the high polarizability alkoxy group, shows the highest clearing point and maximum Δ*n*. Meanwhile, these four new LC mixtures have larger ∆*ε*, lower *γ*_1_/*K*_11_, and comparable Δ*n* than those of the parent mixture **HTD**. The lateral fluorine substituent on the second benzene ring (**T_1_**) brings out a larger dipole moment than that on the first benzene ring (**T_2_**); accordingly, the ∆*ε* value of the corresponding LC mixture **H_1_** is bigger than that of mixture **H_2_**. The two lateral fluorine substituents on the benzene rings of the LC diluter bring out a larger dipole moment; thereby, the ∆*ε* of the corresponding LC mixture **H_4_** is further increased. As we know, the large ∆*n* and the small *γ*_1_/*K*_11_ bring out a large FoM value. The numerical relationships of the FoM values at 25 °C are as follows: **H_1_** > **H_2_** > **H_4_** > **H_3_**. The larger the value is, the better the response performance is. It means that the LC diluter **T_1_** may be more conducive to the fast response time of LC devices at 25 °C. Taking all the collected data at 25 °C into consideration, it is concluded that the diluter molecule **T_1_** could be the most suitable LC dopant for LC mixtures with high ∆*n* and large ∆*ε*.

## 3. Materials and Methods

### 3.1. Materials

We obtained the LC diluter molecules used in this work through three classical organic synthetic reactions including Wittig, substitution, and Sonogashira coupling reactions [35]. The commercial high-∆*n* LC mixture HTD028200-200 was purchased from Jiangsu Hecheng Display Technology Co., LTD (Nanjing, China). Its detailed LC performance parameters include: threshold voltage is 1.93 V, saturation voltage is 2.89 V, appearance is milky liquid, specific resistance ≥ 2 × 10^11^ Ω·cm, clearing point is 93.8 °C, Δ*n* value is 0.27 at 25 °C. The LC diluters were then blended with the parent LC mixture HTD028200-200 to prepare the corresponding LC mixtures, and the doping concentration was kept at 10 wt.%. The liquid crystal cells were purchased from the Northern Liquid Crystal Engineering Research and Development Centre.

### 3.2. Characterization and Measurement

Under the N_2_ atmosphere, the Shimadzu DSC-60 instrument was unitized to measure the clearing points (*Tc*) of all LC mixtures by controlling the heating/cooling rate at 10 °C/min. The low-temperature storing temperature (*Ts*) [36] was measured by a Haier Ultralow Temperature Freezer DW-86W100. The LC diluters **T1**–**T4** were added into the parent LC mixture (HTD028200-200, noted as **HTD**) with a mass fraction of 10%, respectively, to prepare four new LC mixtures **H1**–**H4**. The ∆*n* and *γ*_1_/*K*_11_ values of all LC mixtures were calculated from the measured phase retardation and transient current method [6]. The ∆*ε* values of all LC mixtures were measured with a multifrequency LCR meter HP-4274 [7]. No special instructions; all of the measurements for LC mixtures were carried out at 25 °C, 40 °C, a frequency of 1 kHz, and a wavelength of 633 nm. A Linkam THMS600 hot-stage was used to control the heating/cooling rate when carrying out the experiment.

Equation (1) can be used to deduce the clearing points of the new LC mixtures, which were extrapolated by the host-guest method [37].

(1) $$T_{c}=xT_{c}^{2}+\left(1-x\right)T_{c}^{H}$$

where *x* refers to the doping concentration of the diluter molecule, *T_c_^2^* is the extrapolation value of the clearing point, and *T_c_^H^* is the clearing point of the parent LC mixture. The temperature-dependent birefringence of the LC mixtures can be described as follows [38,39]:

(2) $$∆n=∆n_{0}S=∆n_{0}{\left(1-T/T_{c}\right)}^{\beta }$$

(3) $$∆n=G\frac{\lambda^{2}\lambda^{*2}}{\lambda^{2}-\lambda^{*2}}$$

where ∆*n*_0_ is the birefringence at temperature *T* = 0 K, *S* is the molecular order parameter, *β* is the material constant, *G* is the proportionality constant, and λ* is the average resonance wavelength. The temperature-dependent viscoelastic coefficient of the LC mixtures can be described as the following equation:

(4) $$\frac{\gamma_{1}}{K_{11}}=A\frac{exp\left(E_{a}/k_{b}T\right)}{{\left(1-T/T_{c}\right)}^{\beta }}$$

In Equation (4) [40], A, *k_B_*, and *E_a_* represent the proportionality constant, Boltzmann constant, and activation energy, respectively. The comprehensive performance of each compound was comparatively studied with the FoM parameter, which is defined as the following equation:

(5) $$FoM=\frac{∆n^{2}\cdot K_{11}}{\gamma_{1}}$$

In Equation (5) [31], the large ∆*n* value and the small *γ*_1_/*K*_11_ value achieve the large *FoM* value, which means the better response performance of the LC material.

## 4. Conclusions

We have proposed one effective method to improve the electro-optical properties of high-∆*n* liquid crystal mixtures by using a series of tolane-LC diluters terminated by one difluorovinyl group. Compared with the parent high-∆*n* LC mixture, the corresponding LC mixture optimized by doping one of our LC diluters shows a higher clearing point, a larger dielectric anisotropy, a lower viscoelastic coefficient (decreased by 16.8~28.8%), a higher FoM value, and the equivalent nematic temperature range and birefringence (~0.258), which will improve the response time of LCoS devices in AR displays. Increasing the doping concentration (from 10% to 20%) of our LC diluters in the parent LC mixture further improves the electro-optical properties, while the clearing point of the new LC mixture decreases to below 80 °C. More importantly, the addition of a new LC diluter achieves a perfect balance between high ∆*n*, large ∆*ε*, and low *γ*_1_/*K*_11_ of high-∆*n* LC mixtures, which completely makes up for the shortcomings which existed in previously reported LC diluters. The widespread applications of these LC diluters in emerging LC optical devices are foreseeable. Our research results lay a theoretical foundation and experimental data for the development of fast-response LC mixtures for AR displays.

## Figures and Tables

**Table 1:** The phase transition temperature of LC mixtures.

LC Mixtures	Diluter Structures	*T_c_* (°C)	*T_c_*^1^ (°C) ^a^	*T_c_*^2^ (°C) ^b^	Nematic Range (°C)
**HTD**	—	93.8	—	—	108.8
**H_1_**	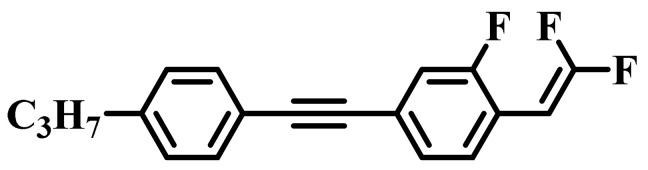	89.5	43.46	50.8	104.5
**H_2_**	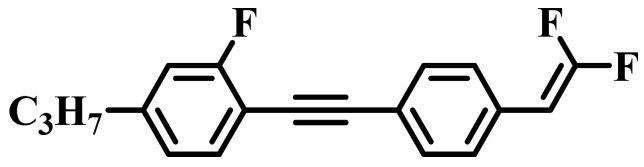	91.1	61.84	66.8	106.1
**H_3_**	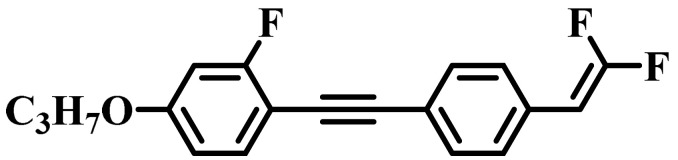	93.9	103.51	94.8	108.9
**H_4_**	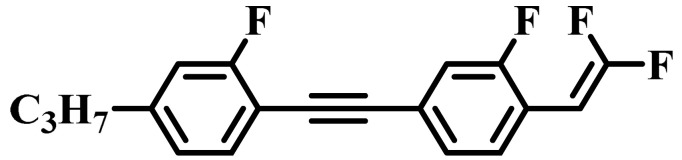	86.6	28.29	21.8	101.6

^a^*T*_C_^1^ is the diluter clearing point temperature from the DSC instrument measurement. ^b^ *T*_C_^2^ is the value extrapolated from the parent LC mixture HTD based on 10% molar concentration with the guest-host method.

**Table 2:** The measured physical properties of these LC mixtures.

LC Mixtures	∆*n* (*λ* = 633 nm)		∆*n_0_*	*β*	*G* (µm^−2^)		λ* (µm)	
	25 °C	40 °C			25 °C	40 °C	25 °C	40 °C
**HTD**	0.262	0.252	0.339	0.158	2.46	2.35	0.287	0.288
**H_1_**	0.252	0.242	0.322	0.144	2.50	2.54	0.283	0.277
**H_2_**	0.254	0.247	0.312	0.119	2.53	2.72	0.283	0.271
**H_3_**	0.258	0.250	0.327	0.141	2.59	2.61	0.281	0.277
**H_4_**	0.248	0.236	0.309	0.129	2.50	2.39	0.282	0.281

**Table 3:** Parameters related to the visco-elastic coefficient of LC mixtures.

LC Mixtures	*γ*_1_/*K*_11_ (ms/µm^2^)		A	*E_a_* (meV)
	25 °C	40 °C		
**HTD**	16.09	10.20	3.71 × 10^−4^	268.3
**H_1_**	11.22	7.73	4.17 × 10^−4^	257.7
**H_3_**	13.39	8.91	5.61 × 10^−4^	254.4
**H_2_**	12.32	8.59	1.35 × 10^−4^	230.6
**H_4_**	12.09	7.84	6.04 × 10^−4^	250.1

**Table 4:** Measured physical properties of LC mixtures at 25 °C.

Code	H_1_	H_2_	H_3_	H_4_
Diluter structures	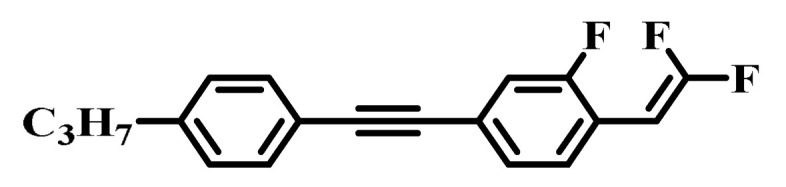	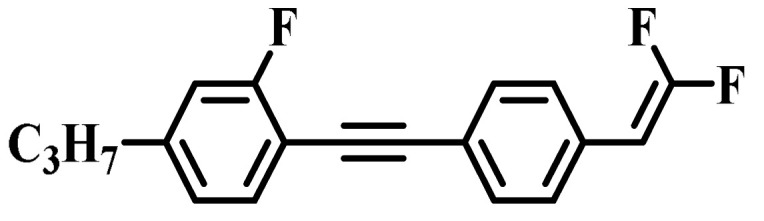	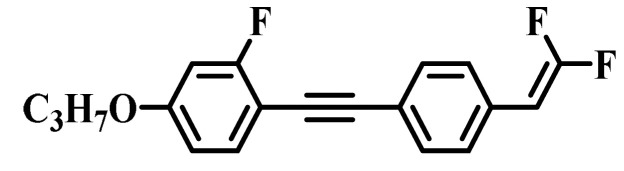	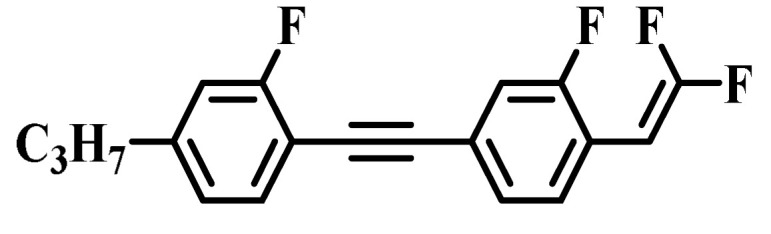
T_s_ (°C)	<−15	<−15	<−15	<−15
T_c_ (°C)	89.5	91.1	93.9	86.6
∆*n*	0.252	0.254	0.258	0.248
∆*ε*	7.7009	7.4413	7.6410	7.9759
*γ*_1_/*K*_11_ (ms/µm^2^)	11.22	13.39	12.32	12.09
*FoM* (μm^2^/s)	5.66	5.24	4.97	5.09

## Data Availability

The data presented in this study are available in this article.

## Sample Availability

Samples of the compounds **T_1_**–**T_4_** are available from the authors.
